# Supplementary material for: The invasion phenotypes of glioblastoma depend on plastic and reprogrammable cell states
Source: Nat Commun. 2025 Jul 19;16:6662. doi: 10.1038/s41467-025-61999-1 (PMC12276355; doi:10.1038/s41467-025-61999-1)
Supplement: Supplementary file 1 — Supplementary Information [file 41467_2025_61999_MOESM1_ESM.pdf]

Milena Doroszko, Rebecka Stockgard, et al.: The invasion phenotypes of glioblastoma depend on plastic and reprogrammable cell states

Supplementary figures and tables

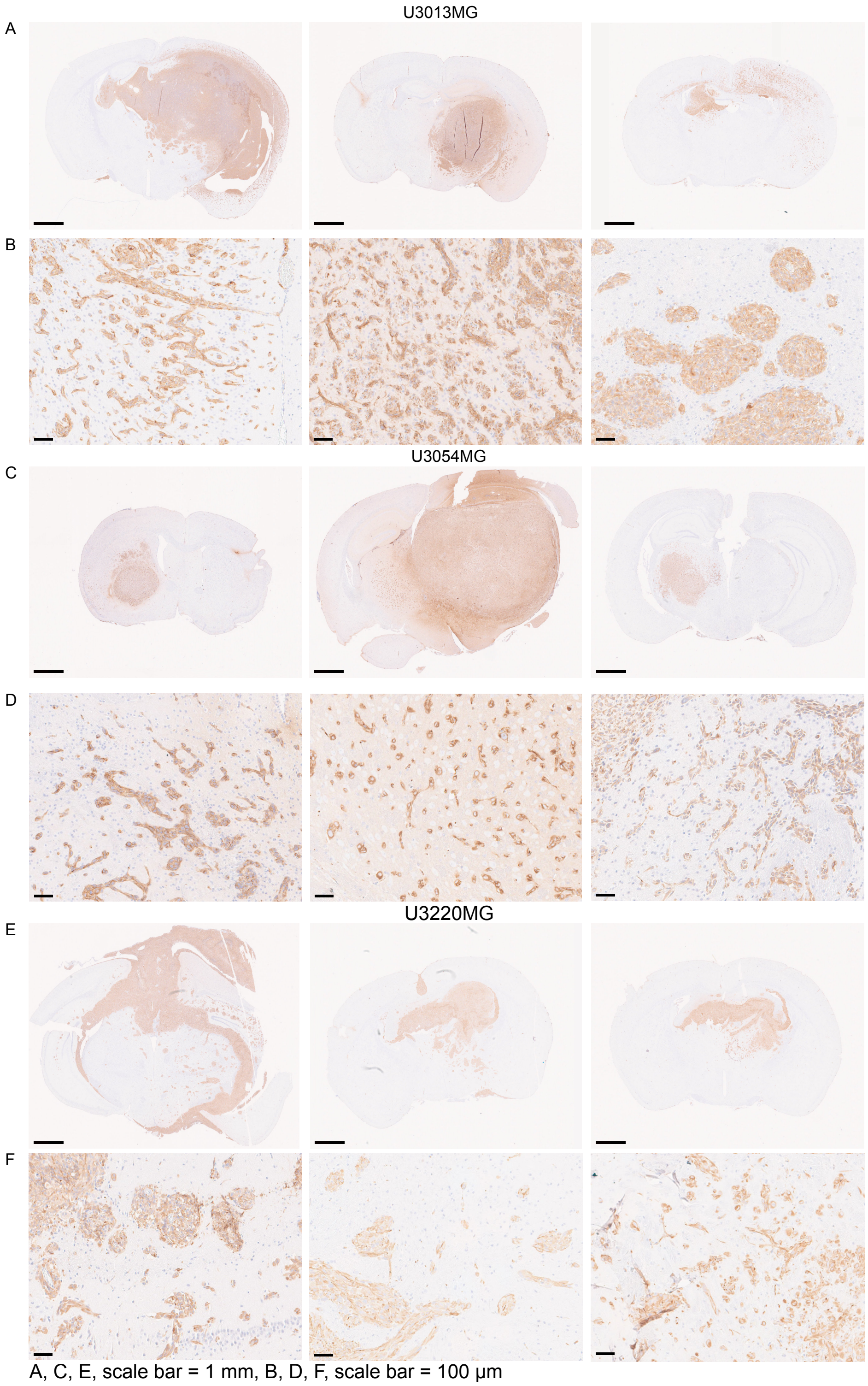

Supplementary Figure 1: Representative growth phenotypes in mice orthotopically grafted with different primary glioblastoma lines. (A) U3013MG (B) Detailed view of invasion phenotype of U3013MG (C) 3054MG (D) Detailed view of invasion phenotype of U3054MG (E) 3220MG (F) Detailed view of invasion phenotype of U3220MG. (A, C, E) Scale bar indicating 1mm. (B, D, F) Scale bar indicating 100 μm. Representative sections shown for n=10 independent mouse replicates for each PDCX model shown.

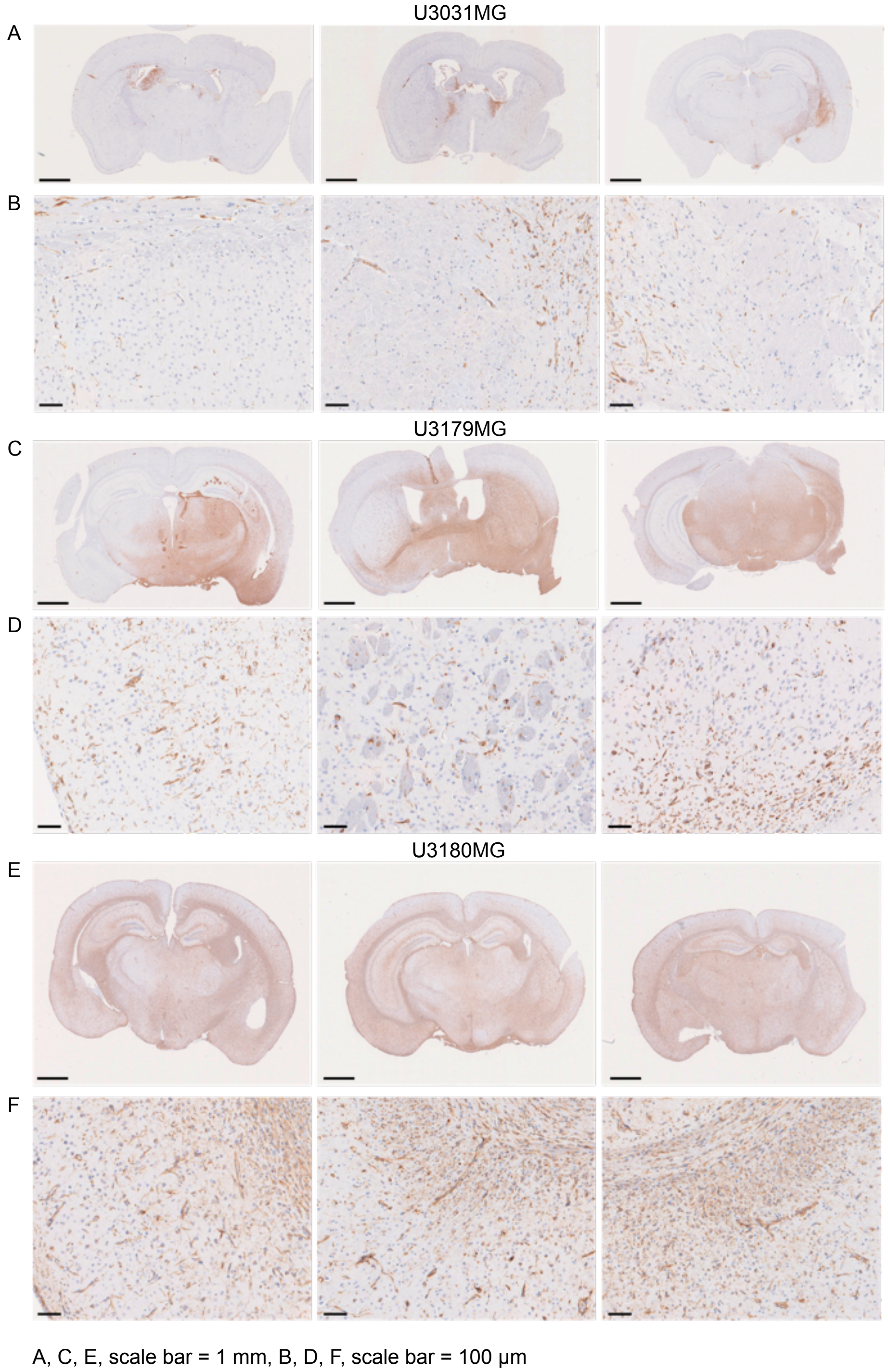

Supplementary Figure 2: Representative growth phenotypes in mice orthotopically grafted with different primary glioblastoma lines (A) U3031MG (B) Detailed view of invasion phenotype of U3031MG (C) 3179MG (D) Detailed view of invasion phenotype of U3179MG (E) 3180MG (F) Detailed view of invasion phenotype of U3180MG. (A, C, E) Scale bar indicating 1mm. (B, D, F) Scale bar indicating 100 µm. Representative sections shown for n=10 independent mouse replicates for each of PDCX model shown.

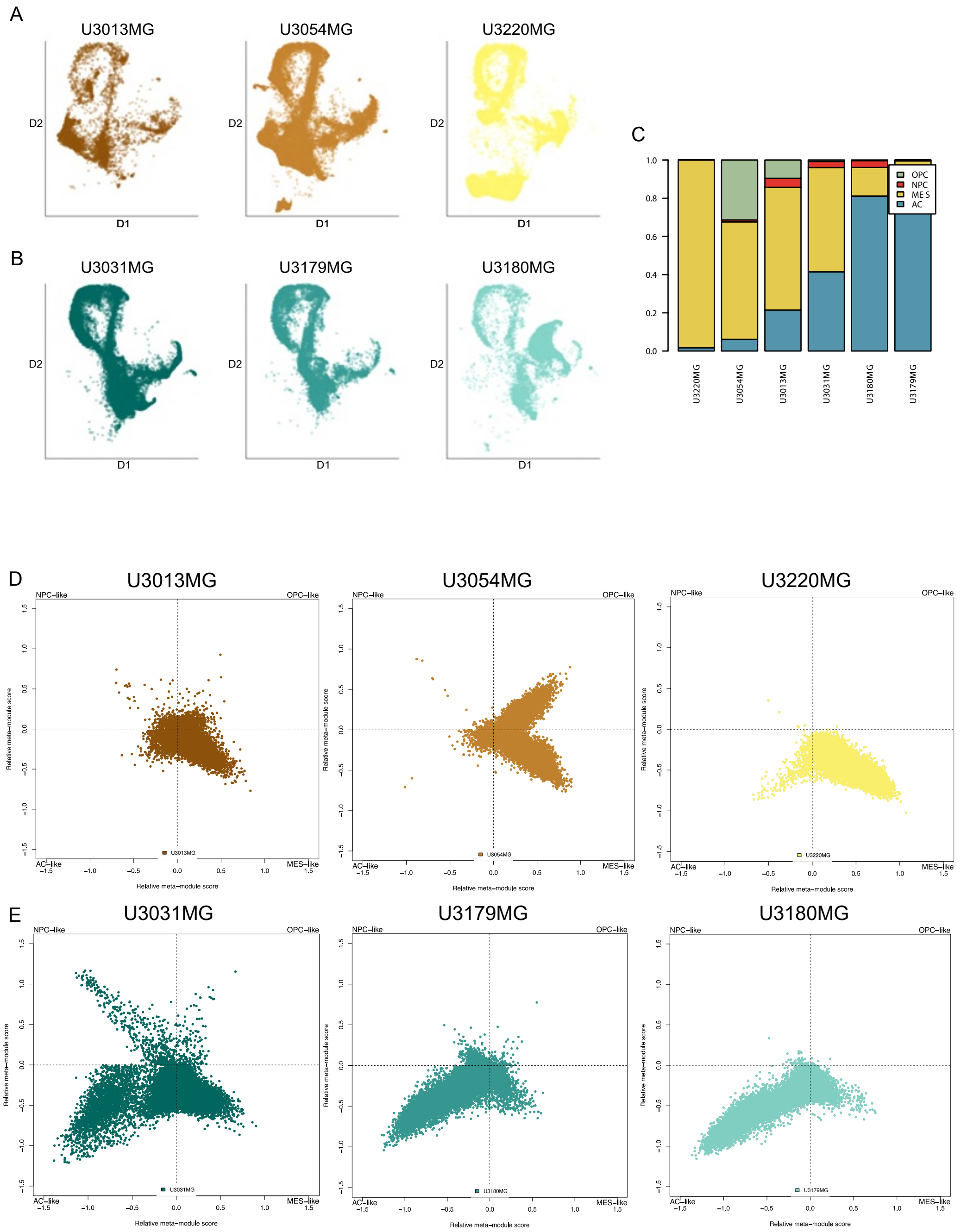

Supplementary Figure 3: UMAP projections and cell state plots for each separate sample in Figure 2 of the main manuscript. (A) UMAP displaying perivascular invading, bulk forming cell lines. (B) UMAP displaying diffuse growing cell lines. (C) Stacked bar plots displaying the abundance of cells in different cell-states. (D,E) Individual cell-state plots of all cell lines. (n=1 *in vitro* sample, n=2 *in vivo* sample scRNAseq runs for each of the 6 GBM lines, except U3031MG, and U3179MG which were run as n=1 *in vitro* sample, n=2 *in vivo* sample; total of 119,766 cells transcriptomes)

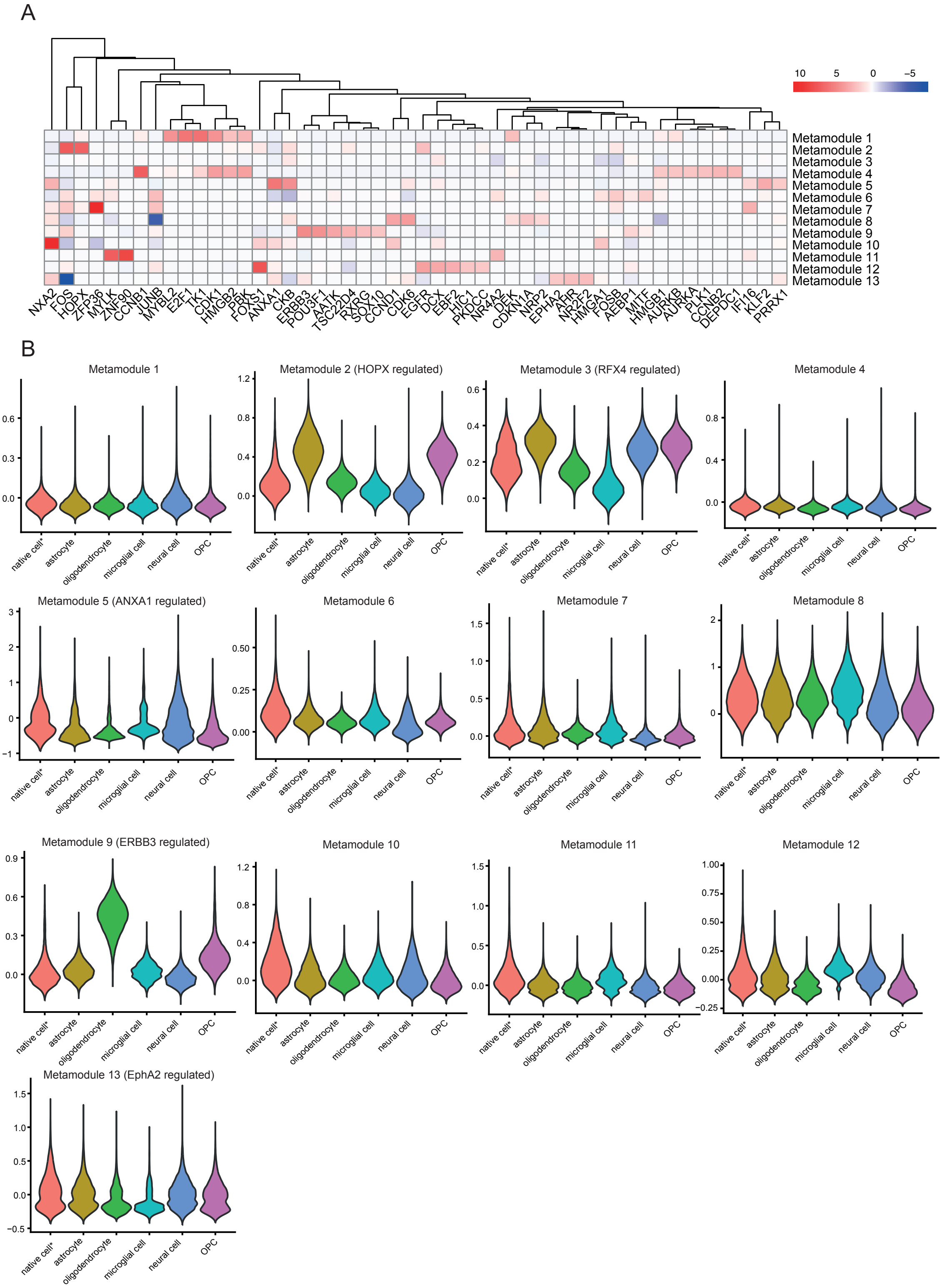

*annotation provided with data distribution, corresponding to vascular and unmarked cells in original publication.

Supplementary Figure 4: scregclust metamodules. (A) Regulator assignments for each metamodule, relating to Figure 3 of the main manuscript. (B) enrichment of metamodule signatures in cells from [1], separated by annotated cell type. The underlying data has n=1 *in vitro* sample, n=2 *in vivo* sample scRNAseq runs for each of the 6 GBM lines, except U3031MG, and U3179MG which were run as n=1 *in vitro* sample, n=2 *in vivo* sample; total of 119,766 cells transcriptomes.

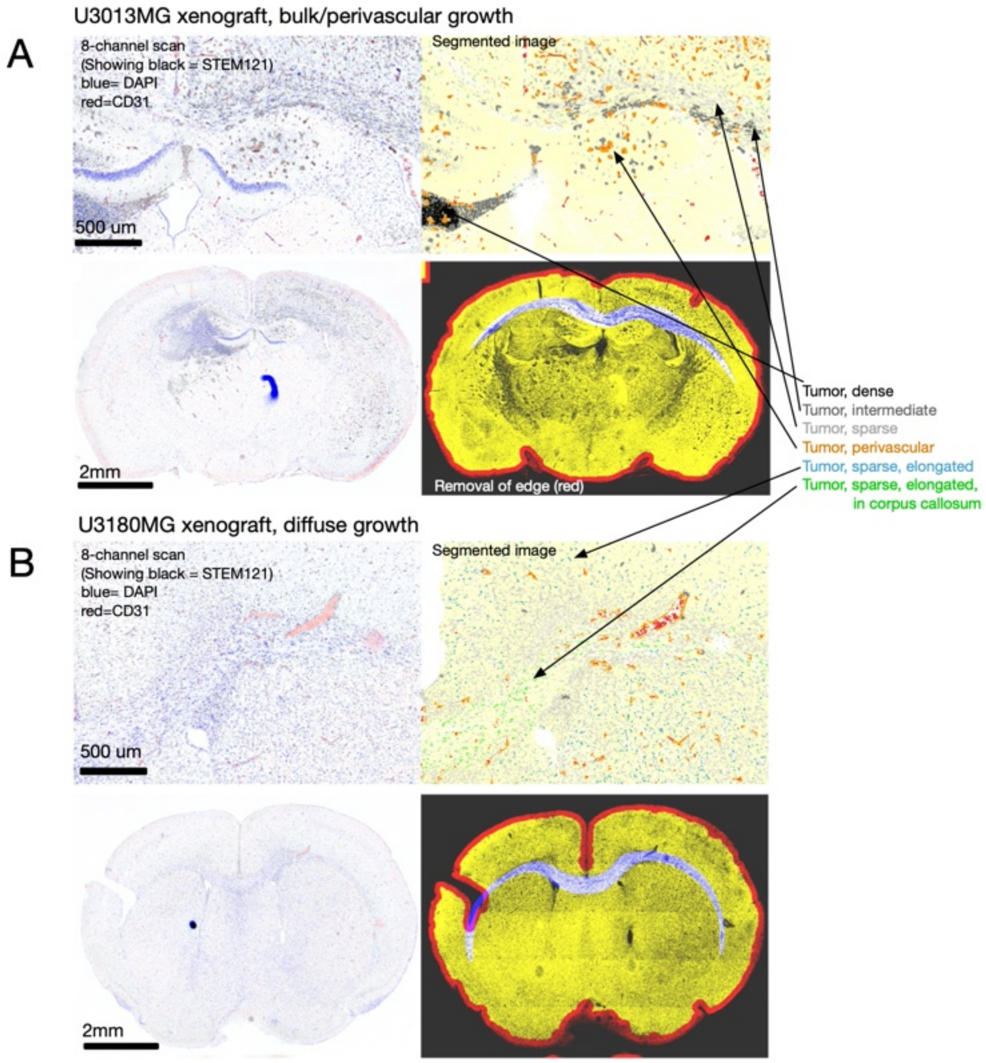

Supplementary Figure 5: Image Segmentation: (A) U3013MG example; (B) U3180MG example. Our image segmentation pipeline is implemented in Matlab (code available upon request). In essence, it loads a QPTIFF file representing the scan of multispectral protein staining. The pipeline begins by performing a crude segmentation using imsegkmeans (Matlab) to identify tumor, vascular, brain, and glass background pixels. Subsequently, a series of morphological operations are applied to identify each of the 9 classes described in the main manuscript. Tumor cells are delineated by watershed segmentation and assigned classes 1, 2, and 3 (as detailed in Figure 4 of the paper) based on their density. Additionally, cells are categorized as perivascular (class 4 in the main paper) if they are within 10 pixels (20 microns) of a blood vessel, as bundled tumor cells (class 5) if they are arranged in a circular pattern, detected by a Laplacian-of-Gaussian filter, and as diffuse invading cells (class 6 and 7) if they are located in low-density areas and have an eccentricity greater than 0.9. Class 6 cells are situated within the corpus callosum (manually defined for each scan), while class 7 cells are outside of the corpus callosum. Classes 8 and 9 represent blood vessels and background brain, respectively, as defined by the original imsegkmeans clustering. See also Figure 4. Representative sections shown for n=10 independent mouse replicates for each of the 2 PDCX models shown.

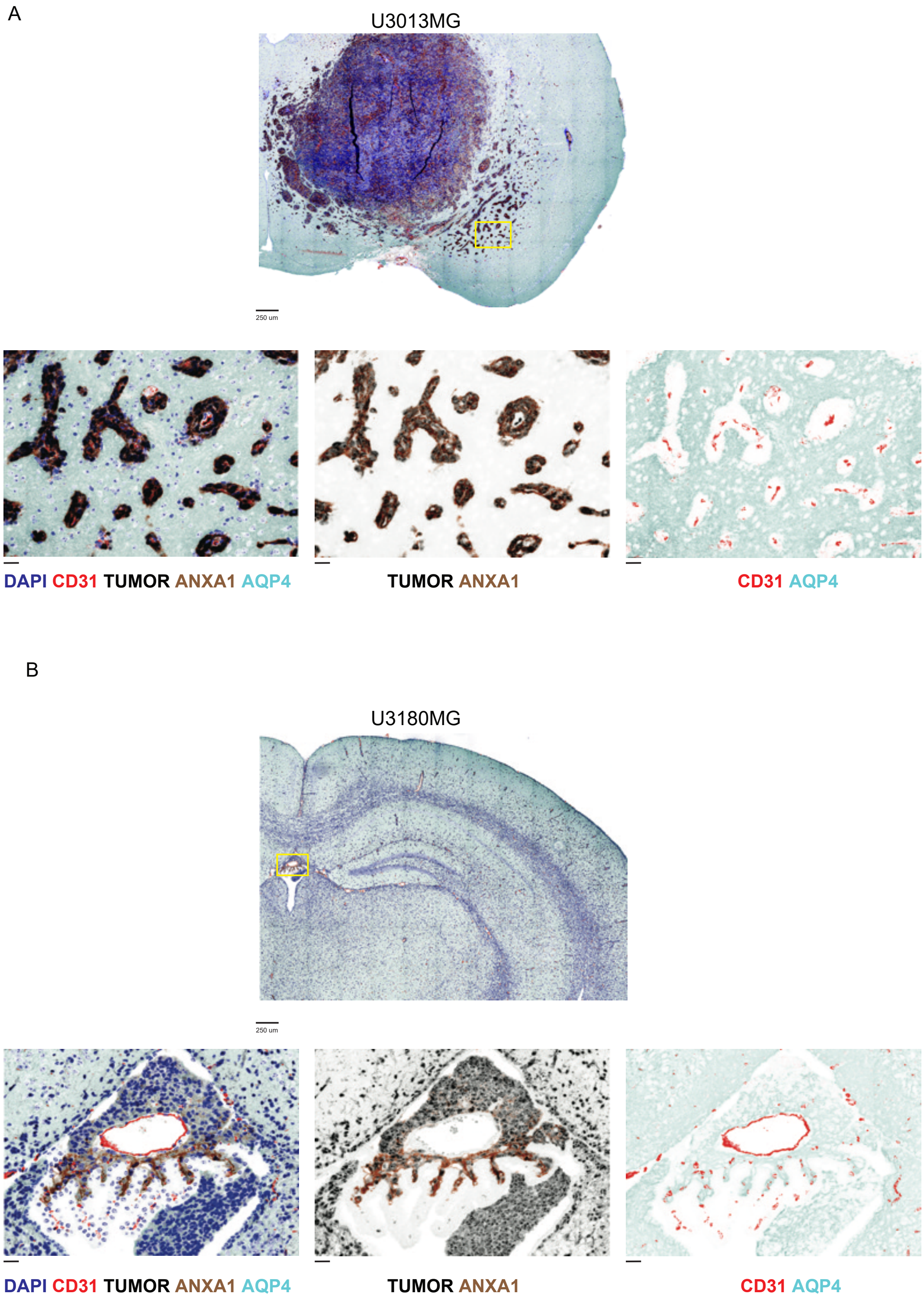

Supplementary Figure 6: Multispectral staining of U3013MG and U3180MG xenografts to display perivascular invasion phenotype. CD31 (red) marks the blood vessels, STEM121 (black) marks the tumor cells, AQP4 (cyan) marks the astrocytic end-feet, ANXA1 (brown) to show ANXA1 expression. Scale bars for the zoomed in images indicate 20 um. Representative sections shown for n=10 independent mouse replicates for each of the 2 PDCX models shown.

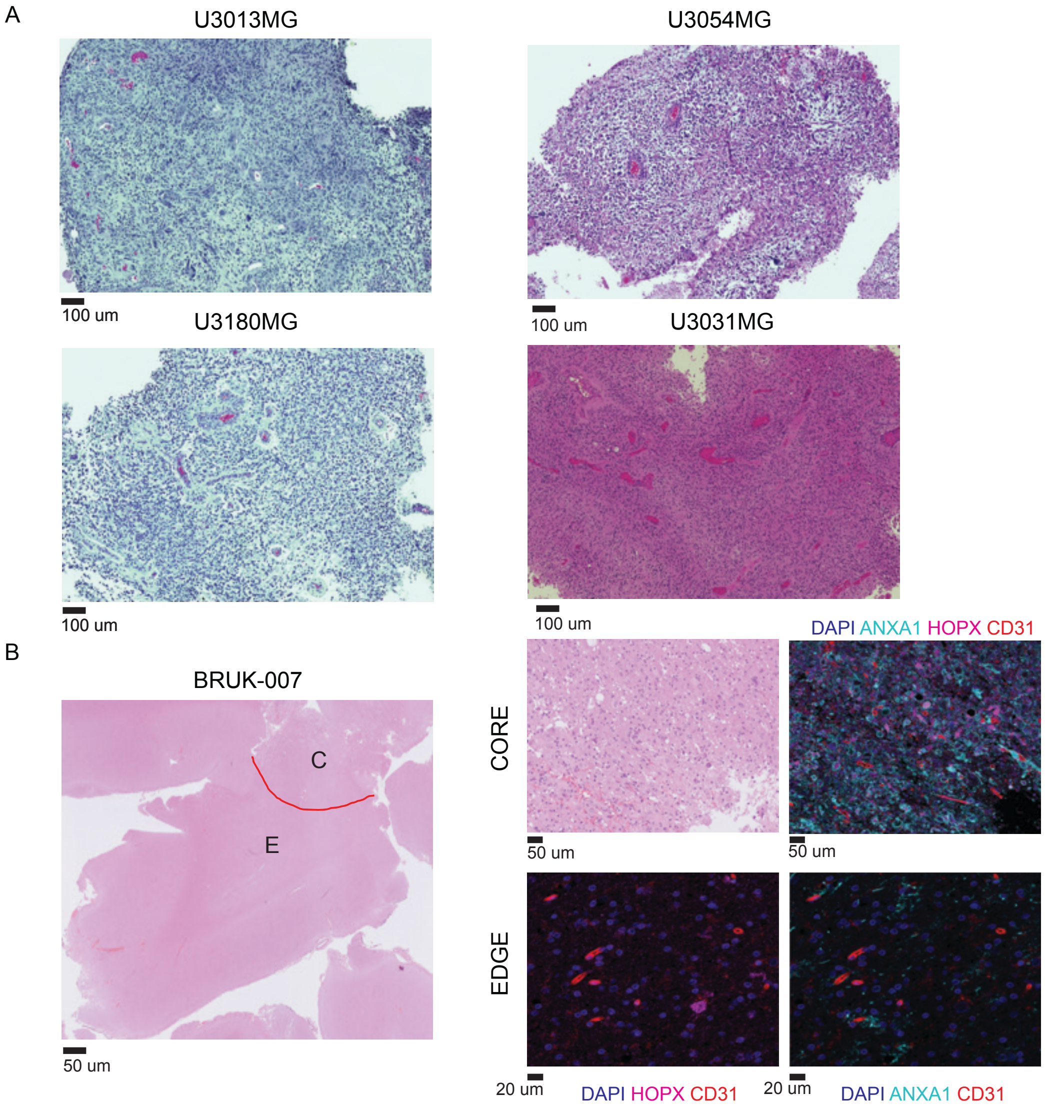

Supplementary Figure 7: Histology examples. (A) H&E staining of patient tissue. Scale bar indicating 100 μm. Representative sections shown for n=10 independent mouse replicates for each of the 2 PDCX models shown. (B) Patient from BrainUK cohort. H&E staining of patient tissue. C indicates the tumor core region and E the leading edge region. Right panel shows the accompanying multispectral stainings for ANXA1 and HOPX. Representative section selected from tumor sections of tumor samples from n=14 patients.

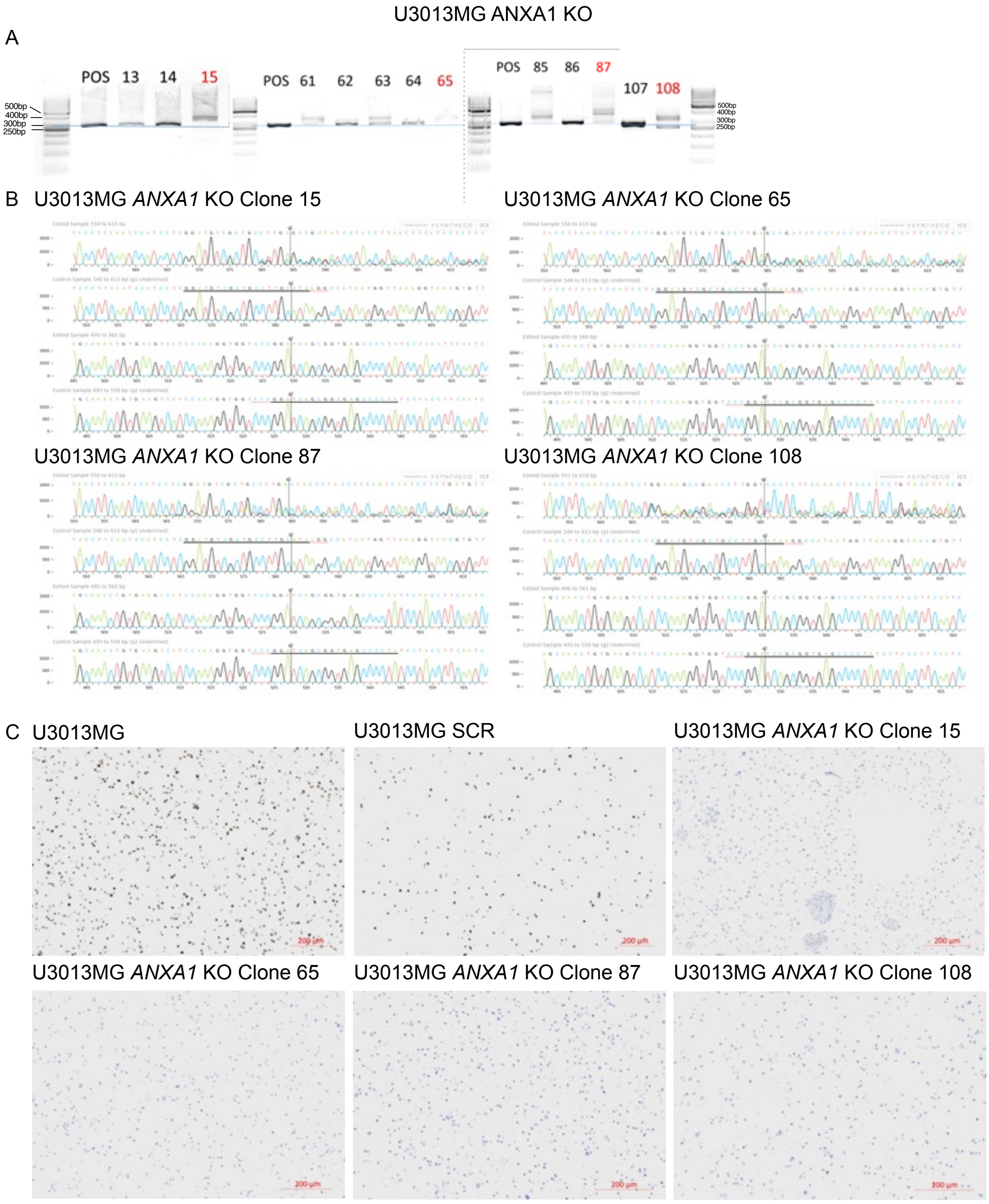

D

Client Sample Name	U3013-lucp28	3013SCR	3013ANXA1KO-MIX1
Sample Code	CL00002002	CL00009638	CL00009639
D8S1179	13,14	13,14	13,14
D21S11	31.2,32.2	31.2,32.2	31.2,32.2
D7S820	9,11	9,11	9,11
CSF1PO	10,12	10,12	10,12
D3S1358	17,19	17,19	17,19
TH01	6,9,3	6,9,3	6,9,3
D13S317	14,14	14,14*	14,14
D16S539	10,10	10,10	10,10
D2S1338	25,26	25,26	25,26
D19S433	14,14	14,14	14,14
vWA	16,16	16,16	16,16
TPOX	8,8	8,8	8,8
D18S51	13,14	13,14	13,14
AMEL	X,X	X,X	X,X
D5S818	12,12	12,12	12,12
FGA	18,24	18,24*	18,24

Supplementary Figure 8: Knockout confirmation, ANXA1. (A) Agarose gel of PCR results for the targeted ANXA1 region, with alterations in clones with index 15, 65, 87, and 108. Expected amplicon size is 281 bp. (B) Sanger sequencing confirmation of knockout in each clone. (C) ANXA1 protein staining for the source culture, scramble control (SCR) and KO clones. Representative images from n=2 independent stainings and 10 inspected microscopic fields for each staining. (D) STR profiling of the 1:1:1:1 mixture of the knockout clones which was used for the mouse experiments. See also Figure 6.

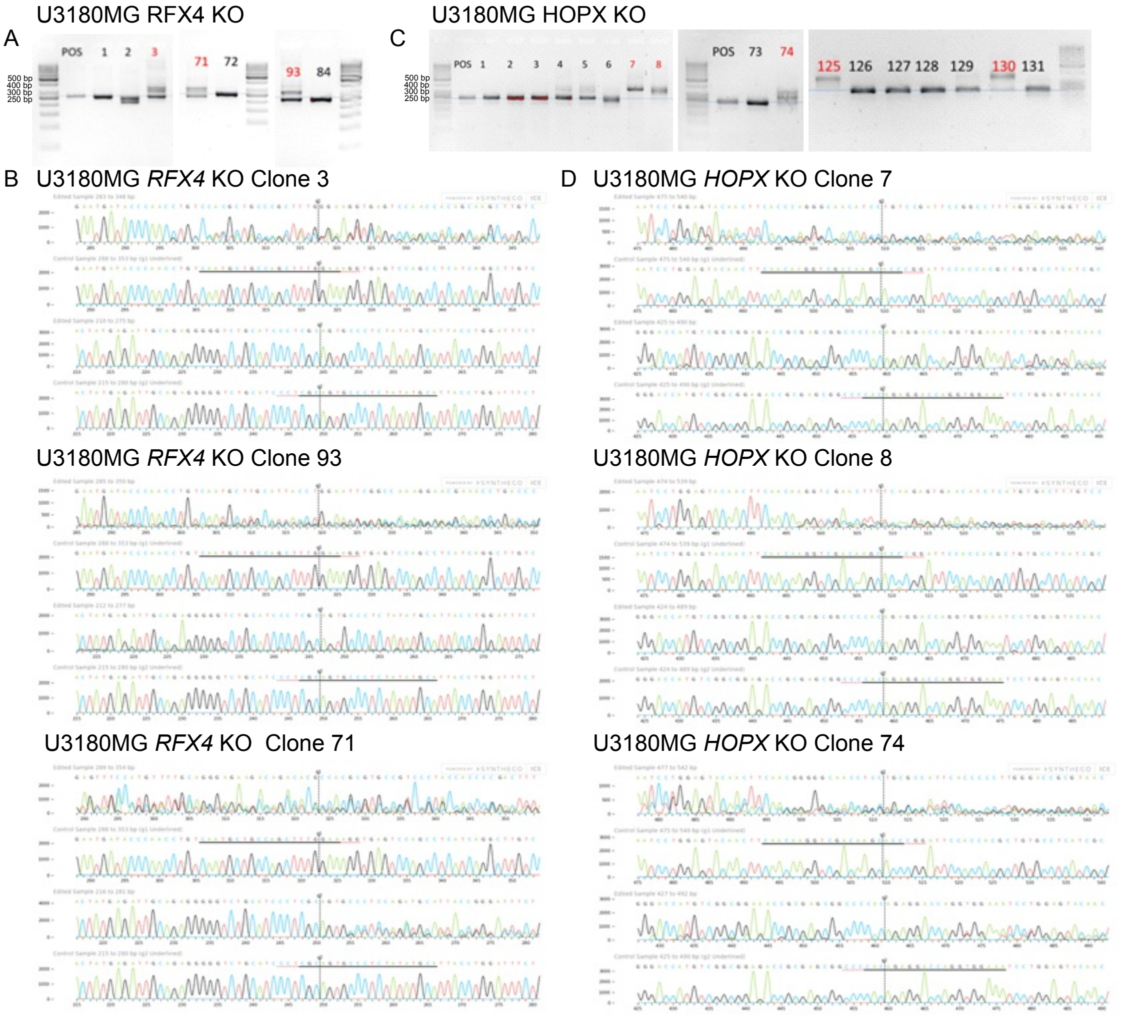

E

Client Sample Name	U3180-luc p26	3180 SCR	3180 HOPX-KO	3180 RFX4KO-MIX1
Sample Code	CL00002006	CL00009644	CL00009646	CL00009642
D8S1179	13,14	13,14	13,14	13,14
D21S11	30,30	30,30	30,30	30,30
D7S820	10,11	10,11	10,11	10,11
CSF1PO	10,12	10,12	10,12	10,12
D3S1358	15,17	15,17	15,17	15,17
TH01	6,9.3	6,9.3	6,9.3	6,9.3
D13S317	8,11	8,11	8,11	8,11
D16S539	9,13	9,13	9,13	9,13
D2S1338	20,25	20,25	20,25	20,25
D19S433	12,13	12,13	12,13	12,13
vWA	17,18	17,18	17,18	17,18
TPOX	8,9	8,9	8,9	8,9
D18S51	15,16	15,16	15,16	15,16
AMEL	X,Y	X,Y	X,Y	X,Y
D5S818	10,11	10,11	10,11	10,11
FGA	22,24	22,24	22,24	22,24

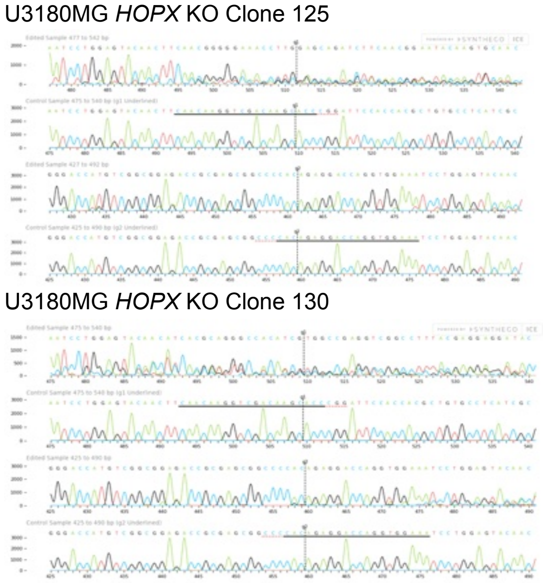

Supplementary Figure 9: Knockout confirmation. (A) Agarose gel of PCR results for the targeted RFX4 region, with alterations in clones with index 3, 71, 93. The band of interest is 249 bp. (B) Sanger sequencing confirmation of knockout in each clone. HOPX (C) Agarose gel of PCR results for the targeted HOPX region, with alterations in clones with index 7, 8, 74, 125, 130. The band of interest is 226 bp. (D) Sanger sequencing confirmation of knockout in each clone. (E) STR profiling of the uniform mixture of the knockout clones which was used for the mouse experiments. See also Figure 6.

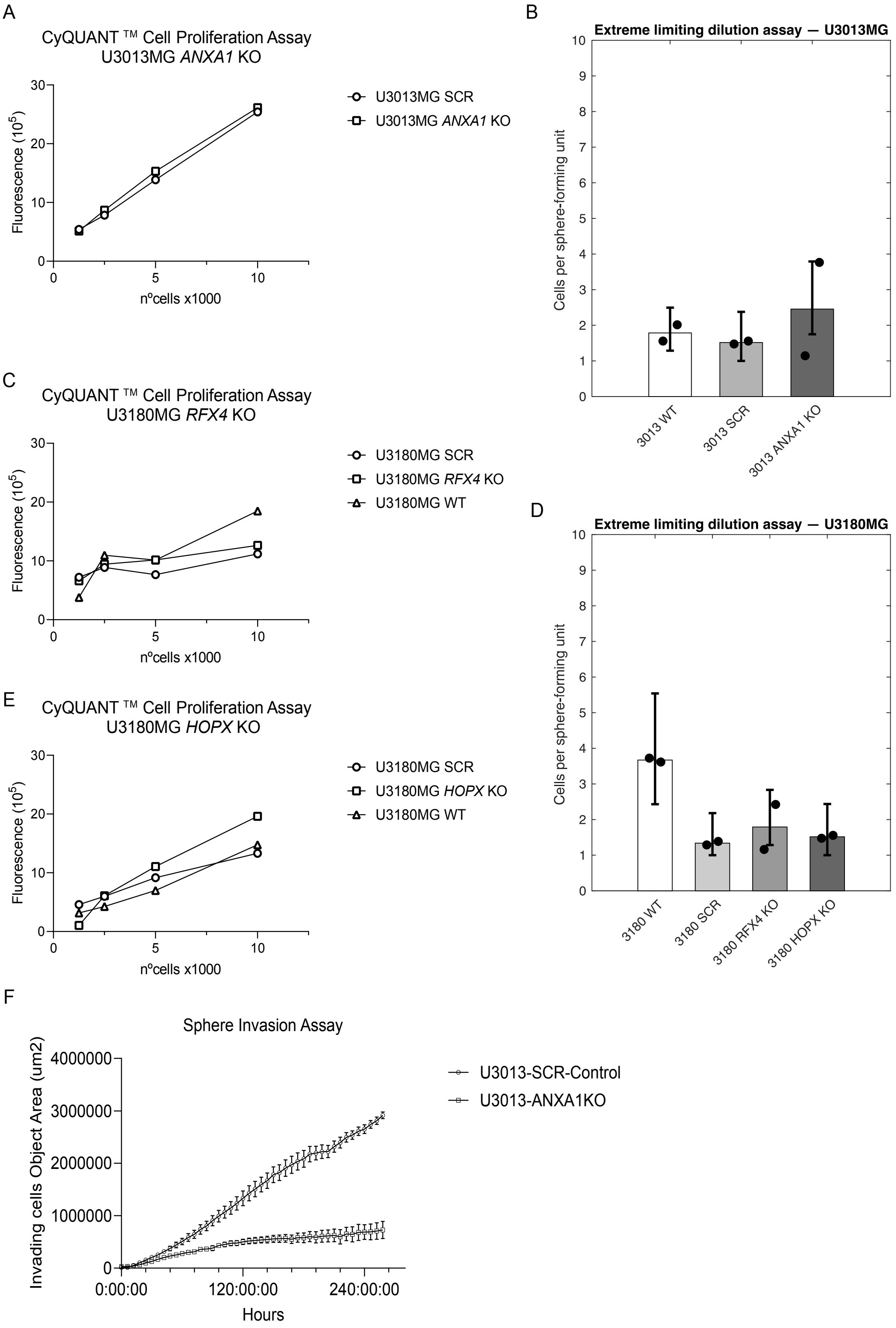

Supplementary Figure 10: In vitro characterization of glioblastoma cells with knockout of ANXA1, RFX4, and HOPX. (A) Cell proliferation assay for U3013MG SCR and U3013MG *ANXA1* KO. (B) Extreme limiting dilution assay for U3013MG, U3013MG SCR, and U3013MG *ANXA1* KO. N=12 replicate wells per dose level, in two replicate 8-dose series (estimates for each series shown as points; bars are 95% confidence intervals). (C) Cell proliferation assay for U3180MG, U3180MG SCR and U3180MG *RFX4* KO. (D) Cell proliferation assay for U3180MG, U3180MG SCR and U3180MG *HOPX* KO. (E) Extreme limiting dilution assay for U3180MG, U3180MG SCR, and U3180MG *RFX4* KO, and U3180MG *HOPX* KO. (F) Sphere invasion assay for U3013-SCR and U3013-ANXA1KO. All experiments were independently repeated twice.

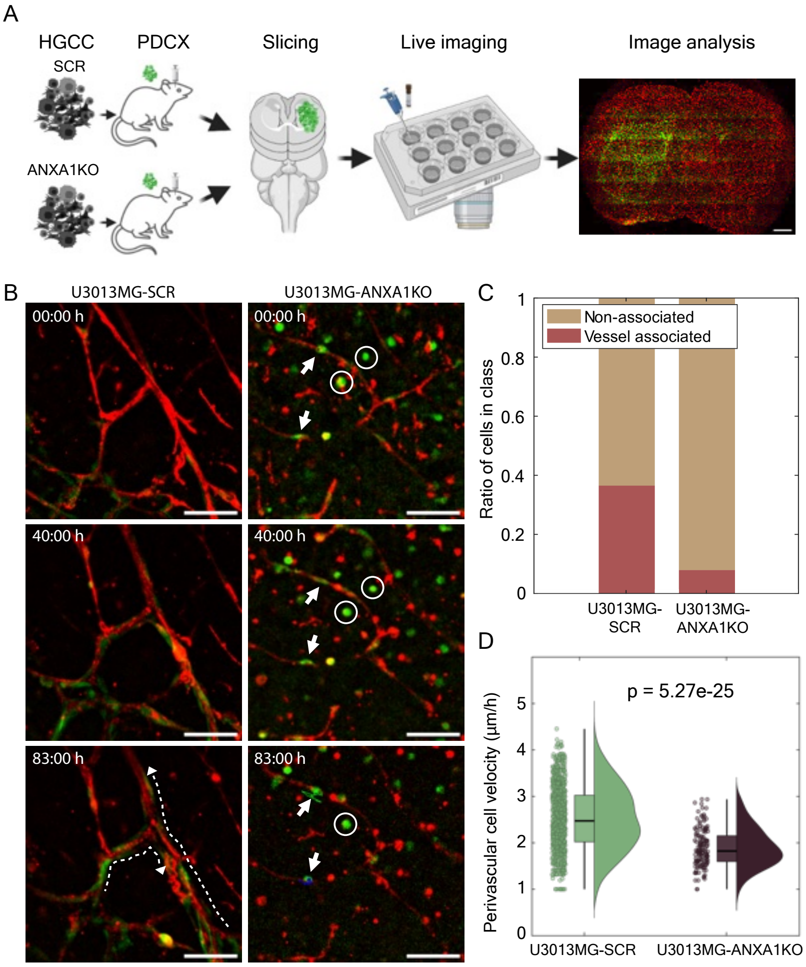

Supplementary Figure 11: Ex vivo brain slice assay for U3013-SCR and U3013-ANXA1KO. (A) Graphical demonstration of the experimental pipeline. Created in BioRender. Nelander, S. (2025) https://BioRender.com/z7km6i0 (B) Still images from ex vivo brain slices comparing U3013-SCR migrating along blood vessels and U3013-ANXA1-KO moving more diffusely. Scale bar is 100 micron. Representative fields-of-view from a total of n=34 selected regions. (C) Quantification of cells' vessel association. (D) Velocity of the cells that are associated with the vasculature. Both (C) and (D) are based on n=4 brain slices and 34 counted regions in ANXA1 knockout and n=3 brain slices and 31 regions of interest in control. Total of 3497 cells tracked.

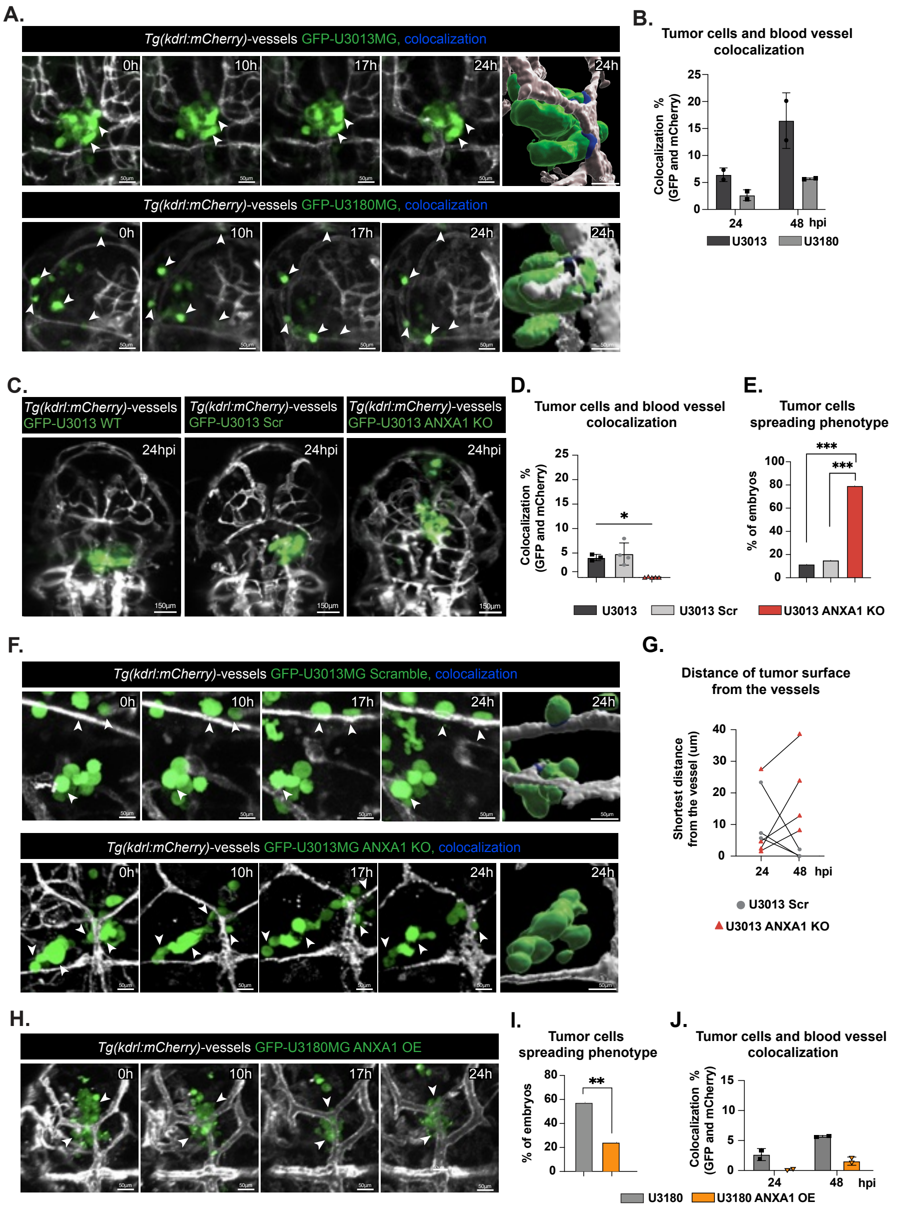

Supplementary Figure 12: ***ANXA1*** **modulates tumor–vessel interactions in vivo.** (A) Confocal time-lapse imaging of U3013 and U3180 tumor cells (GFP, green) in zebrafish with vasculature labeled by *Tg(kdrl:mCherry)* (white), imaged at 0, 10, 17, and 24 hours post injection (hpi). Right panels: 3D renderings of tumor (green), vessels (white), and colocalized volume (blue). n = 2 embryos per cell line, scale bar = 50 μm. White arrowheads mark tumor cells. Representative images from n=4 embryos. (B) Quantification of tumor–vessel colocalization at 24 and 48 hpi for U3013 and 3180 cell lines. (C) Light sheet imaging of U3013, U3013-SCR, and U3013-ANXA1KO cells (green) at 24 hpi; vessels in white. Representative images from n = 3, 4, and 5 embryos, respectively. Scale bar = 150 μm. (D) Quantification of tumor–vessel colocalization (from panel C). One-way ANOVA, *p < 0.01. (E) Percentage of embryos showing diffuse/spreading phenotype at 24 hpi. Fisher's exact test, *** $p < 0.0001$. n = 80 embryos per group. (F) Confocal time-lapse imaging of U3013-SCR and U3013-ANXA1KO cells, as in (A). Representative images from n = 2 embryos per condition. Scale bar = 50 μm. (G) Quantification of tumor–vessel surface distance (from the two embryos in Panel F). n=4 objects in ANXA1 knockout, n=3 objects in scramble control. (H) Still snapshots from confocal time-lapse imaging of U3180-ANXA1OE cells at 0, 10, 17, and 24 hpi. Representative images from n = 2 embryos. Scale bar = 50 μm. White arrowheads mark tumor cells. (I) Percentage of embryos showing diffuse/spreading phenotype at 24 hpi. Fisher's exact test, p < 0.0001. n = 75 embryos per group. (J) Quantification of tumor–vessel colocalization at 24 hpi in U3180 and U3180-ANXA1OE cells. n = 2 and n = 2 embryos, respectively. Scale bar = 50 μm.

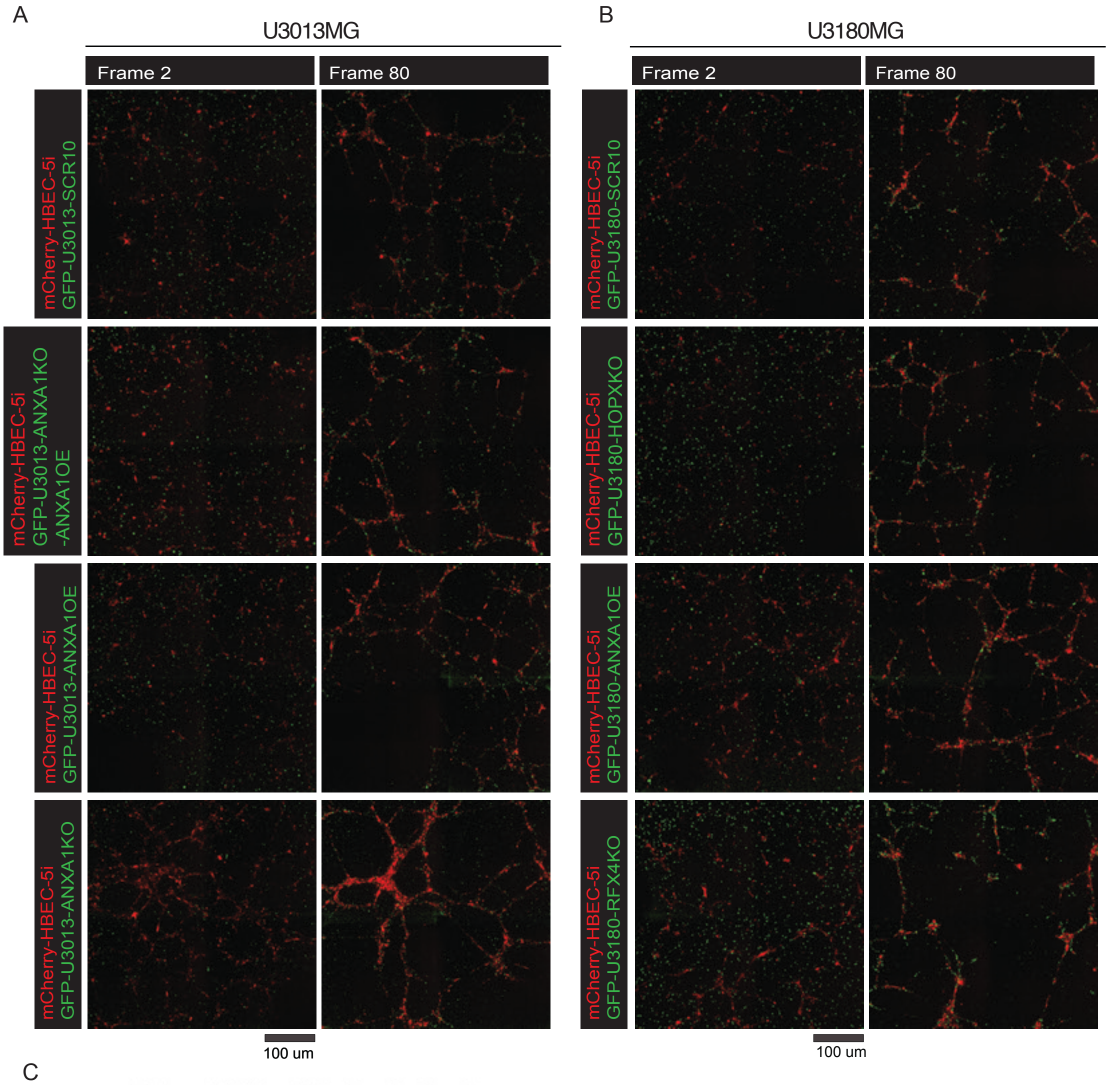

C

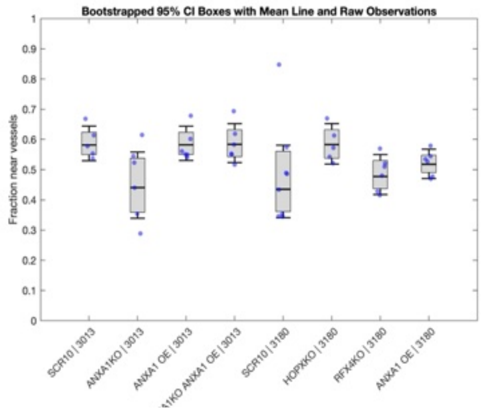

Cell Line	Mean Fraction	CI_Low	CI_High	p value
U3013-SCR	0.581514774	0.549395	0.6238995	NaN
U3013-ANXA1KO	0.440569594	0.358915	0.538052	0.001
U3013-ANXA1OE	0.58189716	0.550285	0.623732	0.512
U3013-ANXA1KO-ANXA1OE	0.583711546	0.543218	0.6322011	0.528
U3180-SCR	0.435079184	0.360863	0.560735	NaN
U3180-HOPXKO	0.582896042	0.538193	0.6322663	0.021
U3180-RFX4KO	0.477238166	0.43749	0.5300171	0.197
U3180-ANXA1OE	0.517716567	0.490443	0.5476867	0.085

Supplementary Figure 13: In vitro 3D Co-culture assay with tumor cells and endothelial cells. (A) Stills from co-culture assay comparing U3013-SCR, ANXA1KO-ANXA1OE, ANXA1OE, ANXA1KO. (B) Stills from co-culture assay comparing U3180-SCR, HOPXKO, ANXA1OE and RFXKO. Representative images from 5-7 co-culture assays per condition. (C) Fraction of GFP-positive tumor cells near blood vessels under each condition. Boxes show 25-27 percentile bootstrap distribution, error bars are the 95% bootstrap confidence interval. n=5-7 coculture assays per condition, total of 48 coculture assays.

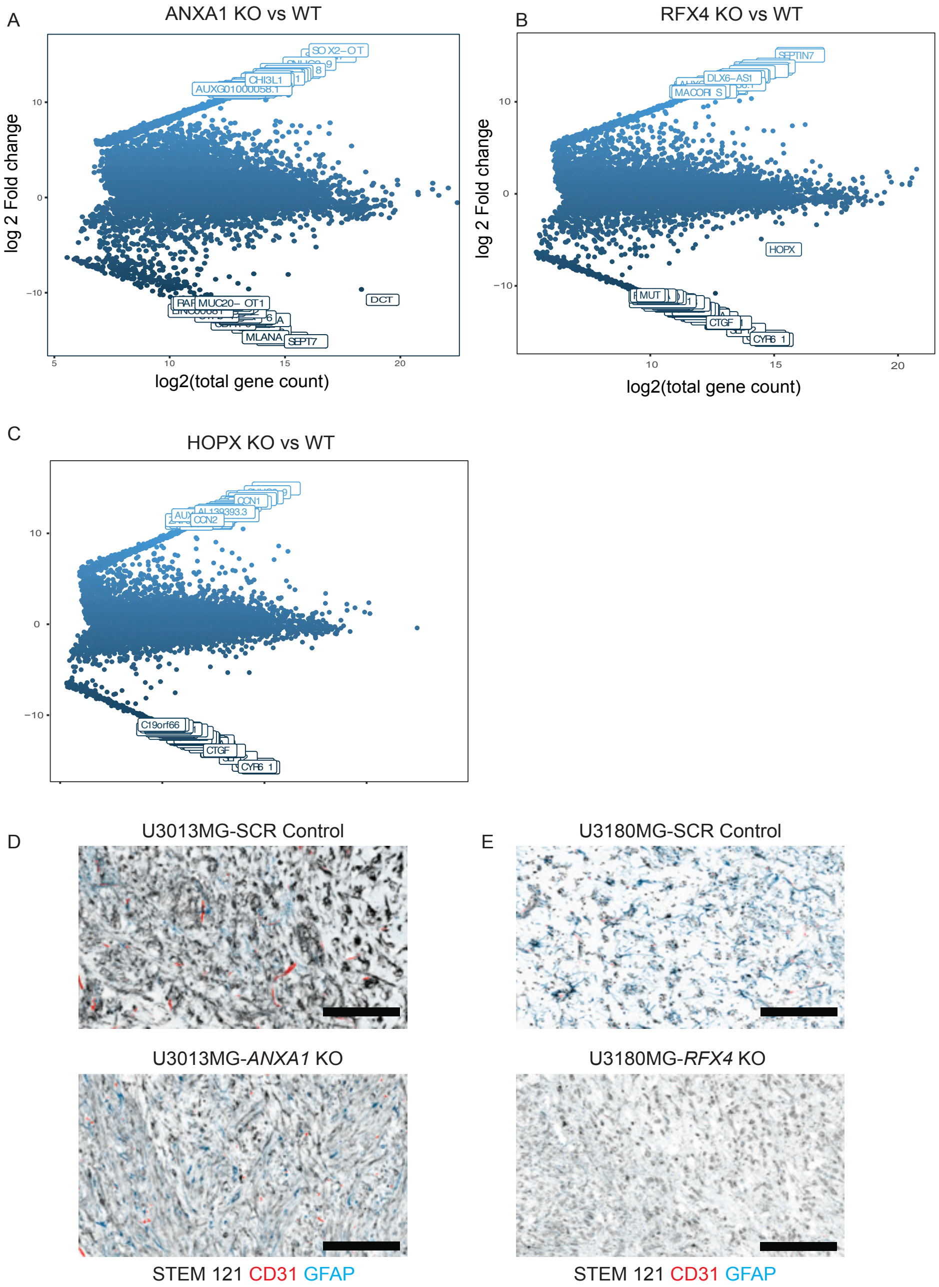

Supplementary Figure 14: Differentially expressed genes. (A) Detected by scRNAseq of ANXA1 knockout U3013MG PDCXs compared to U3013MG (scramble gRNA) controls (n=2 scRNAseq runs, 12069 cells). (B) RFX4 knockout U3180MG PDCXs (n=2 scRNAseq runs, 10824 cells). (C) HOPX knockout U3180MG PDCXs compared to U3180 (scramble gRNA) controls (n=2 scRNAseq runs, 13270 cells). See also Figure 7. (D, E) GFAP stainings for ANXA1 and RFXKO, representative images selected from a total of 3 mice. Scale bar = 100 micron.

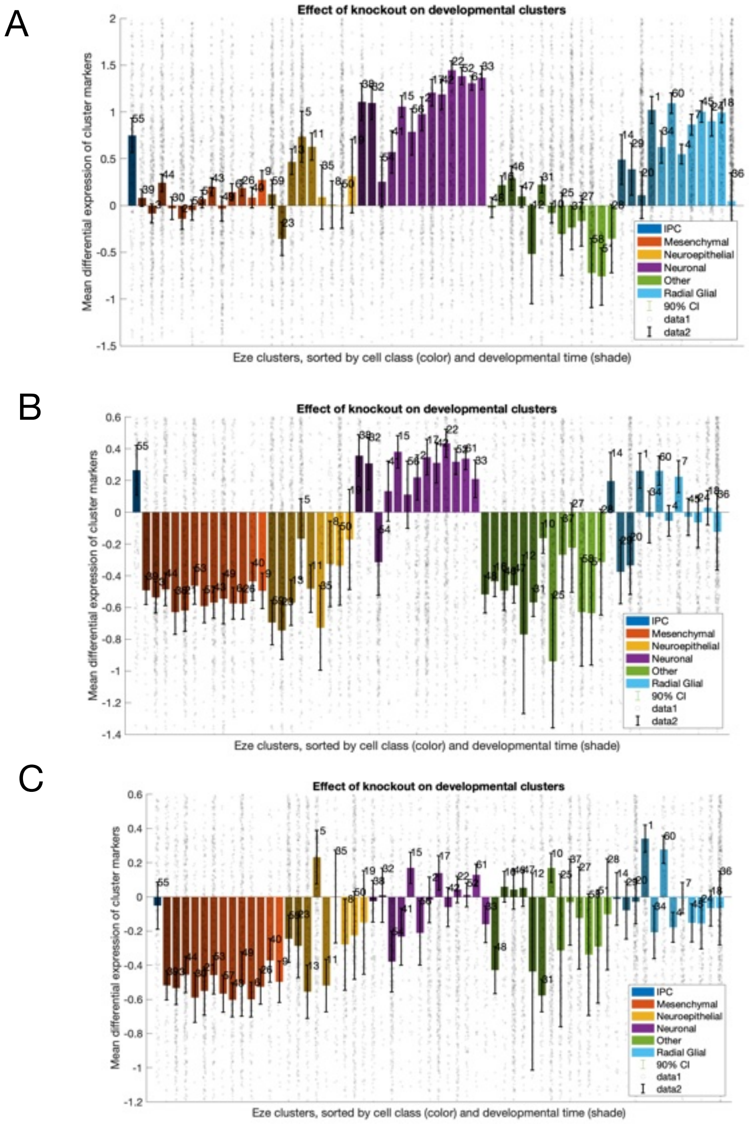

Supplementary Figure 15: Projection of knock-out results on the Eze et al atlas of the developing human brain.. Differentially expressed gene log2 fold change values for each gene were mapped onto the 61 sets of cluster markers reported by [2]. The bars show the average log2 fold change for each cluster, with clusters sorted by each cluster's annotation (cell type) and the average developmental stage of the cells forming that cluster. Error bars are 95 % confidence intervals. Each of the 61 clusters contain an average of n=282 genes (shown as light grey points), ranging between n=34 (minimum) and n=1111 (maximum)

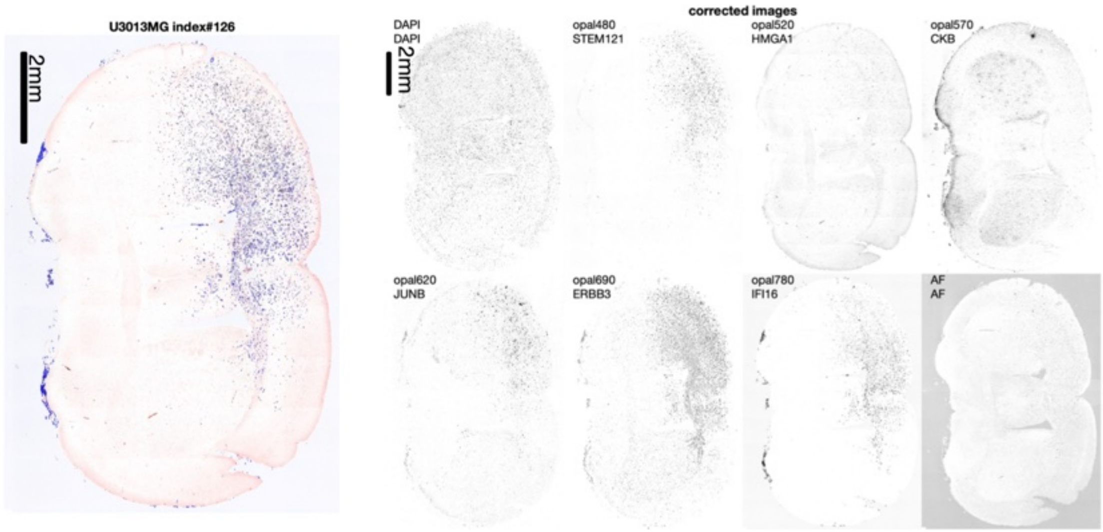

Supplementary Figure 16: ERBB3 positive U3013MG cells in the corpus callosum. The picture shows immunofluorescence scans of a representative U3013MG xenograft (chosen from 10 independent replicate mice) with antibodies against the STEM121 antigen (human-specific antibody, left picture), and proteins HMGA1, CKB, JUNB, ERBB3, IRF16. DAPI denotes nuclear staining and AF denotes the autofluorescence channel. Note ERBB3 positive cells in the corpus callosum (asterisk).

Lentiviral vectors

Cas9 D10A nickase (blasticidine antibiotic selection) VB210424-1001zhs	pLV[Exp]-EF1A>Cas9(D10A)-hPGK>Bsd
Dual guide RNA vector h*HOPX* (neomycine antibiotic selection)	pLV[Exp]-U6>hHOPX[gRNA\#1]U6>hHOPX[gRNA\#2]-CMV>Luciferase-hPGK>Neo
Dual guide RNA vector h*ANXA1* (neomycine antibiotic selection)	pLV[Exp]-Neo-U6>hANXA1[gRNA\#1] U6>hANXA1[gRNA\#2]-CMV>Luciferase
Dual guide RNA vector for h*RFX4* (neomycine antibiotic selection)	pLV[Exp]-Neo-U6>hRFX4[gRNA\#1] U6>hRFX4[gRNA\#2]-CMV>Luciferase
Dual guide RNA vector for scramble guides	pLV[Exp]-Neo-U6>Scramble[gRNA\#1]-U6>Scramble[gRNA\#2]-CMV>Luciferase

Supplementary Figure 17: Structure of lentiviral vectors used for gene targeting.

Dual guide RNA sequences	
HOPX (VB210427-1008ksp)	sRNA\#1 5'-CAACAAGGTCGACAAGCACC-3'
	gRNA\#2 5'-TTTCCACCTGGTCCTCTGTG-3'
ANXA1 (VB211222-1210tgs)	gRNA\#1 5'- GGATGTCGCTGCCTTGCATA-3'
	gRNA\#2 5'-TAGGGGCTCACCGCTGATCC-3'
RFX4 (VB211223-1160cnk)	gRNA\#1 5'-CAATGCTGCCAGCTTTGGAA-3'
	gRNA\#2 5'- TGCATATAGAGGGCACTGCG-3'
Scramble (VB210415-1142zna)	gRNA\#1 5'- GTTCAGGATCACGTTACCGC- 3'
	gRNA\#2 5'- GTTCAGGATCACGTTACCGC- 3'

Supplementary Figure 18: Guide RNAs used for gene targeting.

Gene	Purpose	Fv/Rv	Sequence	Tm	Length (bp)
ANXA1	Genotyping (qPCR)	Fv	GCCTGGTTTATTGAAAATGAAGAGC	60	281
ANXA1	Genotyping (qPCR)	Rv	TCTGAAATGTCCAGGAGGGAACT	60	281
ANXA1	Sanger sequencing (PCR)	Fv	TGGCATATGGTGGGTGGTCTAC	65	938
ANXA1	Sanger sequencing (PCR)	Rv	GGCCCTCAGTGTTGCAGTGT	65	938
HOPX	Genotyping (qPCR)	Fv	GACCGCCTTCCTTCGCT	59	226
HOPX	Genotyping (qPCR)	Rv	GGG TCT CCT CCT CGG AAA G	59	226
HOPX	Sanger sequencing (PCR)	Fv	ACCCTCGCGATCTGTCAAGTC	65	873
HOPX	Sanger sequencing (PCR)	Rv	GACTGGTGACCGAGGGGTTT	65	873
RFX4	Genotyping (qPCR)	Fv	GCCCATCTCTGGAGGACTAGAAAG	60	249
RFX4	Genotyping (qPCR)	Rv	GTGGTAGGGAGGACAAGCCTGAT	60	249
RFX4	Sanger sequencing (PCR)	Fv	TAGCCTGGTGTCATACCCAAAGG	65	748
RFX4	Sanger sequencing (PCR)	Rv	GGACTCCCACACCTCCAATCT	65	748

Supplementary Figure 19: PCR primers.

References

[1] Velmeshev, D. *et al*. Single-cell analysis of prenatal and postnatal human cortical development. *Science* **382**, eadf0834 (2023).

[2] Eze, U., Bhaduri, A., Haeussler, M., Nowakowski, T. & Kriegstein, A. Single-cell atlas of early human brain development highlights heterogeneity of human neuroepithelial cells and early radial glia. *Nat Neurosci* **24**, 584–594 (2021).
