# Supplementary material for: The invasion phenotypes of glioblastoma depend on plastic and reprogrammable cell states
Source: Nat Commun. 2025 Jul 19;16:6662. doi: 10.1038/s41467-025-61999-1 (PMC12276355; doi:10.1038/s41467-025-61999-1)
Supplement: Supplementary file 2 — Description of Additional Supplementary Information [file 41467_2025_61999_MOESM2_ESM.pdf]

Description of Additional Supplementary Files

File Name: Supplementary Data 1
Description: Phenotypic scores for mice injected with U3013MG, U3054MG, U3220MG, U3179MG, U3180MG, and U3031MG.

File Name: Supplementary Data 2
Description: scregclust results.

File Name: Supplementary Data 3
Description: Vectra Polaris panels, list of antibodies used.

File Name: Supplementary Data 4
Description: p values from the survival analysis.

File Name: Supplementary Data 5
Description: Differentially expressed genes (Figure 7).

File Name: Supplementary Data 6
Description: TMA staining results for ANXA1, HOPX, and RFX4 for the HGCC cohort.

File Name: Supplementary Movie 1
Description: Time lapse confocal imaging of mouse brain slices, showing movement of tumor cells in *ANXA1* wild type vs *ANXA1* knockout U3013MG glioblastoma cells. Green=tumor cells marked with green fluorescent protein. Red=blood vessels counterstain with fluorescent dye conjugated lectin.

File Name: Supplementary Movie 2
Description: Time lapse imaging of zebrafish embryonic brain, showing movement and perivascular association of GFP-tagged U3013MG glioblastoma cells.

File Name: Supplementary Movie 3
Description: Time lapse imaging of zebrafish embryonic brain, showing movement and perivascular association of GFP-tagged U3180MG glioblastoma cells.

File Name: Supplementary Movie 4
Description: Time lapse imaging of zebrafish embryonic brain, showing movement and perivascular association of GFP-tagged U3013MG glioblastoma cells with *ANXA1* knockout.

File Name: Supplementary Movie 5
Description: Time lapse imaging of zebrafish embryonic brain, showing movement and perivascular association of GFP-tagged U3013MG glioblastoma cells with *ANXA1* over-expression.
