# Supplementary material for: The invasion phenotypes of glioblastoma depend on plastic and reprogrammable cell states
Source: Nat Commun. 2025 Jul 19;16:6662. doi: 10.1038/s41467-025-61999-1 (PMC12276355; doi:10.1038/s41467-025-61999-1)
Supplement: Supplementary file 14 — Reporting Summary [file 41467_2025_61999_MOESM14_ESM.pdf]

nature portfolio

Corresponding author(s): Sven Nelander
Last updated by author(s): Jun 17, 2025

Reporting Summary

Nature Portfolio wishes to improve the reproducibility of the work that we publish. This form provides structure for consistency and transparency in reporting. For further information on Nature Portfolio policies, see our Editorial Policies and the Editorial Policy Checklist.

Statistics

For all statistical analyses, confirm that the following items are present in the figure legend, table legend, main text, or Methods section.

n/a	Confirmed	
☐	☒	The exact sample size (*n*) for each experimental group/condition, given as a discrete number and unit of measurement
☐	☒	A statement on whether measurements were taken from distinct samples or whether the same sample was measured repeatedly
☐	☒	The statistical test(s) used AND whether they are one- or two-sided<br>*Only common tests should be described solely by name; describe more complex techniques in the Methods section.*
☐	☒	A description of all covariates tested
☐	☒	A description of any assumptions or corrections, such as tests of normality and adjustment for multiple comparisons
☐	☒	A full description of the statistical parameters including central tendency (e.g. means) or other basic estimates (e.g. regression coefficient) AND variation (e.g. standard deviation) or associated estimates of uncertainty (e.g. confidence intervals)
☐	☒	For null hypothesis testing, the test statistic (e.g. *F*, *t*, *r*) with confidence intervals, effect sizes, degrees of freedom and *P* value noted<br>*Give P values as exact values whenever suitable.*
☒	☐	For Bayesian analysis, information on the choice of priors and Markov chain Monte Carlo settings
☒	☐	For hierarchical and complex designs, identification of the appropriate level for tests and full reporting of outcomes
☐	☒	Estimates of effect sizes (e.g. Cohen's *d*, Pearson's *r*), indicating how they were calculated

Our web collection on statistics for biologists contains articles on many of the points above.

Software and code

Policy information about availability of computer code

Data collection	All data was generated by us for this study. More details are provided in materials and methods.
Data analysis	Demultiplexing, counting, and alignment to the human (GRCh38) reference genome were performed using Cell Ranger 3.0.2 (10X Genomics). We performed single-cell analysis using the Seurat package (v. 4) in R. The scRegClust algorithm was used. (github:https://github.com/scmethods/scregclust). For the image analysis the Matlab image analysis toolbox was used which is further described in materials and methods.

For manuscripts utilizing custom algorithms or software that are central to the research but not yet described in published literature, software must be made available to editors and reviewers. We strongly encourage code deposition in a community repository (e.g. GitHub). See the Nature Portfolio guidelines for submitting code & software for further information.

Data

Policy information about availability of data

All manuscripts must include a data availability statement. This statement should provide the following information, where applicable:
- Accession codes, unique identifiers, or web links for publicly available datasets
- A description of any restrictions on data availability
- For clinical datasets or third party data, please ensure that the statement adheres to our policy

Our data has been deposited to GEO (accession ID: GSE270083).

Research involving human participants, their data, or biological material

Policy information about studies with human participants or human data. See also policy information about sex, gender (identity/presentation), and sexual orientation and race, ethnicity and racism.

Reporting on sex and gender	Glioblastoma (GBM) affects all sex and genders with a tendency to be more commonly observed in men. Our models were picked based on their differing growth patterns in mice as representatives of patient GBM. The cell lines used in this study were from 4 female and 2 male patients. U3013MG and U3180MG was studied further in the study and they are from a female and male patient respectively. Although the impact of sex is not investigated further in our study, by studying cell lines from different sexes, we account for the possible biases that can come from sex differences.
Reporting on race, ethnicity, or other socially relevant groupings	Our main cohort is from the HGCC (Human Glioma Cell Culture) Resource. This biobank was collected in Uppsala/Sweden and therefore is primarily made up of Swedish patients. In order to minimize bias that can come from using patients from the same cohort, we have included patient samples from Queen Square/NHNN Repository in figure 5. The ethnic backgrounds of these patients are unknown.
Population characteristics	The median age of diagnosis of GBM is 65, therefore the age of the patients primarily used in this study is all above 60. The rest of the relevant characteristics such as tumor location and mutational status is reported in Figure 1.
Recruitment	The models used in this study are from two biobanks and the tissue were collected and the cell lines were established prior to the start of the study. All samples were collected with the informed written consent of the patients.
Ethics oversight	The collection was approved by the Uppsala Regional Ethical Board, under number 2007/353 for the HGCC biobank and the ethical approval was obtained via BrainUK, ref:21/014 for the Queen Square NHNN Repository.

Note that full information on the approval of the study protocol must also be provided in the manuscript.

Field-specific reporting

Please select the one below that is the best fit for your research. If you are not sure, read the appropriate sections before making your selection.

☒ Life sciences ☐ Behavioural & social sciences ☐ Ecological, evolutionary & environmental sciences

For a reference copy of the document with all sections, see nature.com/documents/nr-reporting-summary-flat.pdf

Life sciences study design

All studies must disclose on these points even when the disclosure is negative.

Sample size	No sample-size calculations were performed to pick the number of cell lines. Patients that represent the invasive phenotypes were chosen due to their consistency as shown in the supplementary table 1.
Data exclusions	The survival data of mice presented indicates the glioma free survival and the mice that did not produce any tumors due to technical errors from injections were excluded.
Replication	The initial characterization of mouse phenotypes were based on n=45 mice (minimum 4 mice per group). For histological analyses, minimum of 3 brains of replicate mice were analyzed per cell line. For the functional in vitro experiments, each experiment was repeated two times.
Randomization	The mice were ordered from the supplier and groups to be injected were determined randomly. Furthermore, 3 independent researchers performed the injections with the same protocol. Further randomization of the experimental groups are not applicable.
Blinding	This pre-clinical study contains in vitro and in vivo oncology experiments. The mice experiments were all done according to strict protocols previously established in the lab. For the analysis of patient samples for figure 5, the pathologist was given different stainings but not the protein information and was only asked to characterize the expression.

Reporting for specific materials, systems and methods

We require information from authors about some types of materials, experimental systems and methods used in many studies. Here, indicate whether each material, system or method listed is relevant to your study. If you are not sure if a list item applies to your research, read the appropriate section before selecting a response.

Materials & experimental systems

n/a	Involved in the study
[ ]	[x] Antibodies
[ ]	[x] Eukaryotic cell lines
[x]	[ ] Palaeontology and archaeology
[ ]	[x] Animals and other organisms
[ ]	[x] Clinical data
[x]	[ ] Dual use research of concern
[x]	[ ] Plants

Methods

n/a	Involved in the study
[x]	[ ] ChIP-seq
[x]	[ ] Flow cytometry
[x]	[ ] MRI-based neuroimaging

Antibodies

Antibodies used	All antibodies used and the relevant information is provided in Supplementary file 3.
Validation	The antibodies were always first validated with immunohistochemistry. For validation, different concentrations of dilutions were used along with different blocking solutions and antigen retrieval buffers. The results were manually analyzed under a microscope. If the protein was interest were expressed in the correct compartment, the best dilution and solutions were picked according to the staining results. For the multi-plex immunofluorescence stainings; the OPAL fluorophores were validated by shuffling the antibody-opal pairs and analyzing the results in order to produce the correct signal.

Eukaryotic cell lines

Policy information about cell lines and Sex and Gender in Research

Cell line source(s)	The cell lines used are from the HGCC biobank. 4 cell lines were from female and 2 cell lines were from male patients. (Detailed information is provided in figure 1) The cell lines investigated further here U3013MG and U3180MG are from female and male patient respectively.
Authentication	All cell lines were STR profiled to authenticate. The report of the STR profiling is provided in the Supplementary figure 6 and 7.
Mycoplasma contamination	All cell lines were routinely tested for mycoplasma infections and confirmed to be negative.
Commonly misidentified lines (See ICLAC register)	NA

Animals and other research organisms

Policy information about studies involving animals; ARRIVE guidelines recommended for reporting animal research, and Sex and Gender in Research

Laboratory animals	For this study, NMRI nude (Foxn1-nu/nu) mice from Janvier Labs and Hsd:athymic nude-Foxn1 mice from Envigo were used. All mice were aged from 6 to 9 weeks old in the beginning of injections. Additionally Tg(kdrl:mCherry) labelled zebrafish embryos were used for additional xenograft generation.
Wild animals	This study did not involve any wild animals.
Reporting on sex	All mice used in this study were female due to their ease of care. Sex was not considered to be factor since the tumor models are not syngenic and are patient-derived.
Field-collected samples	This study did not involve any samples collected from the field.
Ethics oversight	All mouse experiments were conducted in strict accordance with an ethical permit granted by the Uppsala Animal Research Ethical Board, bearing reference numbers C41/14 and 5.8.18-06726/2020.

Note that full information on the approval of the study protocol must also be provided in the manuscript.

Clinical data

Policy information about clinical studies

All manuscripts should comply with the ICMJEguidelines for publication of clinical research and a completedCONSORT checklist must be included with all submissions.

Clinical trial registration	NA
Study protocol	NA

Data collection	NA
Outcomes	NA

Plants

Seed stocks	NA
Novel plant genotypes	NA
Authentication	NA
